# Peptide Receptor Radionucleotide Therapy for Unresectable Paediatric Head and Neck Paraganglioma in Aotearoa New Zealand

**DOI:** 10.1155/crot/9910461

**Published:** 2026-09-07

**Authors:** Matthew McCall, Jonathan Stevenson, Benjamin Chan

**Affiliations:** ^1^ Department of Otolaryngology, Te Whatu Ora Waitaha Canterbury, Christchurch, New Zealand; ^2^ Department of Otolaryngology, Te Whatu Ora Te Toka Tumai, Auckland, New Zealand

**Keywords:** head and neck paraganglioma, paediatric paraganglioma, peptide receptor radionucleotide therapy, PRRT

## Abstract

Head and neck paragangliomas present incidentally or with signs of mass effect. Approximately 30%–40% are hereditary, which typically present at a younger age. Given their proximity to vital structures, they are often inoperable, and alternative treatment options have significant side effects. We present a case of a paediatric patient with a glomus jugulare paraganglioma who received peptide receptor radionucleotide therapy (PRRT), a targeted molecular therapy. We observed a good molecular response stable disease at 12‐month follow‐up and radiologically stable disease at 15 months posttreatment. PRRT is not currently a widely accessible treatment option, and the literature reports a reasonable disease response in treatment of head and neck paraganglioma.

## 1. Introduction

Paragangliomas (PGLs) are neuroendocrine tumours (NETs) derived from paraganglia of the autonomic nervous system [1]. Head and neck PGLs account for approximately 3% of all PGLs [2, 3]. They are typically slow‐growing and painless and are often found incidentally, although they may present with cranial nerve deficits or other signs and symptoms due to mass effect [2, 4–7].

Carotid body tumours are the most common head and neck PGLs, followed by jugulotympanic and vagal tumours [1, 8]. Approximately 30%–40% of PGLs are hereditary, with succinate dehydrogenase (SDH) mutations commonly implicated [9]. Hereditary PGLs typically present at a younger age [10].

Given the proximity of these tumours to vital structures, complete surgical resection is often not feasible [2, 4–7, 11]. In these circumstances, alternative treatment options include external beam or stereotactic radiotherapy, radiofrequency ablation or, rarely, systemic chemotherapy or tyrosine kinase inhibitors [12–14].

Peptide receptor radionucleotide therapy (PRRT) is a targeted molecular therapy. It is a well‐recognised treatment for low‐grade (predominantly gastroenteropancreatic) NET; however, there is increasing evidence supporting its use in higher‐grade NET [15]. PRRT involves chelating a somatostatin peptide analogue, such as DOTATATE, to a therapeutic radioisotope. For example, lutetium‐177, a β‐emitting radioisotope, can be chelated to DOTATATE to form ^177Lu‐DOTATATE (LuTate). This binds to the somatostatin receptor expressed on the tumour cells and may slow cellular replication or cause cell death. Somatostatin receptor expression can be confirmed using a PET DOTATATEscan before initiating treatment [16].

## 2. Case

A 15‐year‐old Māori girl was referred to a head and neck clinic in a public tertiary hospital with headaches, progressive voice changes and diplopia. The patient had no significant medical or surgical history and no family history of note.

On examination, she had a left abducens nerve palsy with diplopia on leftward gaze. She had atrophy of the left side of her tongue with leftward deviation consistent with a left hypoglossal nerve palsy. Flexible nasoendoscopy revealed a left vocal cord palsy. The remaining cranial nerves were examined unremarkably. There were no palpable neck masses.

**FIGURE 1:**
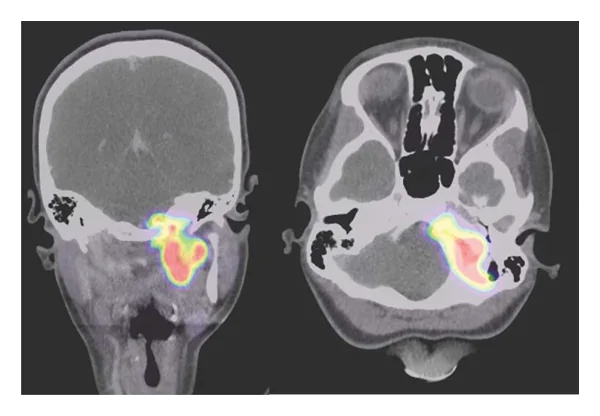
Pretreatment DOTATATE PET‐CT showing an avid left paraganglioma.

An MRI with gadolinium contrast revealed a highly vascular soft tissue mass centred near the left jugular foramen with intracranial extension through the hypoglossal canal and jugular foramen. A DOTATATEPET‐CT confirmed that this mass was DOTATATE‐avid, consistent with a glomus jugulare PGL (Figure 1). The pretreatment standardised uptake value (SUVmax) was 6.9.

Genetic testing identified a pathogenic SDHA variant (heterozygous SDHA: c.91C > T p.(Arg31∗) variant) supportive of a hereditary PGL. Plasma 3‐methoxytyramine levels were elevated, but plasma metanephrines were normal.

After discussion at a regional head and neck multidisciplinary meeting and the National Neuroendocrine Multidisciplinary Team meeting, the patient was deemed to have inoperable disease and was referred for PRRT.

The patient received four PRRT cycles of Lu‐177DOTATATE. Each cycle required a day‐stay admission. A reno‐protective infusion of arginine (25 g) and lysine (25 g) in 1000 mL of 0.9% NaCl was administered before radionucleotide administration by slow intravenous infusion. The cycles were spaced over an 8‐month period. The cumulative dose was 29.5 GBq.

### 2.1. Follow‐Up

During the course of her treatment, the patient experienced improvement in her headaches and diplopia. Her voice remained unchanged.

**FIGURE 2:**
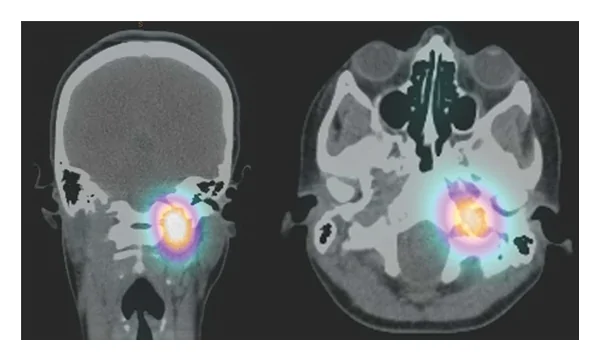
LuTate SPECT‐CT demonstrating good LuTate uptake within left glomus jugulare following the final induction cycle of PRRT.

Repeat DOTATATEPET‐CT at 1 year posttreatment showed stable disease with a reduction in SUVmax from 6.9 to 6.0 (a 13% reduction in activity) and radiological stability on MRI at 15 months posttreatment (Figure 2). She remains symptomatically stable.

## 3. Discussion

This case report describes the use of PRRT to treat a paediatric patient with a hereditary glomus jugulare PGL. In this case, PRRT was recommended due to the potential long‐term adverse effects associated with external beam radiotherapy.

A literature review was completed to investigate the current experience of PRRT use in patients with head and neck PGLs. A search of Medline and the Cochrane database was performed using the following criteria:

1. Prospective studies, retrospective studies or case reports where the participants had been diagnosed with a head and neck PGL.
2. Participants were deemed to have inoperable disease.
3. Participants had completed PRRT, with or without prior treatment.

Five publications were identified meeting the above criteria, including three retrospective studies, one case series and one case report, published between 2012 and 2021. These studies included 35 participants with a mean age of 55 years [2, 4, 6, 7, 11].

Disease response to PRRT is measured by ‘molecular response’ on follow‐up PET‐CT or by ‘radiological response’, based on tumour size, on follow‐up CT or MRI [17]. The definitions of molecular and radiological response are as follows and are generally accepted based on the PERCIST and RECIST criteria, respectively.

Partial molecular response is defined as a reduction of at least 30% in SULpeak (measured by the standardised value uptake [SUVmax] or lean body mass corrected SUV [SULpeak]) of all target lesions detected at baseline. Stable molecular disease is a reduction in SULpeak of less than 30% or an increase of no more than 30% of all target lesions detected at baseline. Partial radiological response is at least a 30% decrease of the sum of maximum diameters of target lesions and no new lesions. Stable radiological response is a reduction in maximum lesion diameter by less than 30% diameter or an increase by no more than 20% [17].

Twenty (57%) cases had at least a partial molecular or radiological response. Twelve (34%) cases had stable disease. Two (6%) cases did not respond to treatment, both of which were carotid body PGLs. One case did not have follow‐up data available. In addition, Estevao et al. reported that those with low radioisotope uptake had less marked response to treatment [2, 4, 6, 7, 11].

One paediatric case was identified—a 14‐year‐old with a carotid body PGL with skeletal metastases [6]. PRRT was initiated for disease progression following prior surgical resection of the primary and photon irradiation of skeletal metastases. Three cycles of PRRT were administered. Four years after completion of treatment, the disease remained radiologically and clinically stable [6].

PRRT does not come without risk. Reported adverse effects include catecholamine crisis and tumour lysis syndrome [18]. In addition, not all PGLs are amenable to PRRT. The tumour must be DOTATATE‐avid, and the rate of DOTATATEuptake in head and neck PGL could be as low as 20% [19].

Other forms of radiotherapy are also used in the management of head and neck PGLs; however, radiotherapy in the head and neck can cause significant short‐ and long‐term adverse effects which include, but are not limited to, xerostomia, trismus, osteoradionecrosis, ototoxicity and lymphoedema [20]. Paulino et al. studied complications in a cohort of children treated with radiotherapy for head and neck rhabdomyosarcoma [21]. All of the 17 who survived to 5‐year follow‐up showed late effects of treatment, including facial growth retardation, neuroendocrine dysfunction and visual and dental problems [21]. Stereotactic radiosurgery has been shown in the literature to be another treatment option with reasonable outcomes; however, this is a specialised form of radiotherapy that is not offered in the public system at the majority of hospitals in New Zealand [13, 14].

These studies suggest that PRRT is a viable treatment for head and neck PGL; however, most cases reported to date have been in older adults. Currently, publicly funded access to PRRT in New Zealand is limited and is only provided in Auckland. Families have to move away from their homes to complete treatment spaced out over a number of months, creating both a psychological and financial barrier.

## 4. Conclusion

The current case report demonstrates an example of PRRT use in head and neck PGL in a paediatric patient resulting in stable disease after 15 months of follow‐up. Prospective research investigating the benefits and risks of PRRT versus current gold‐standard treatment modalities would help to inform future practice.

## Funding

No funding was received for this manuscript.

## Ethics Statement

Single‐patient case reports are outside the scope of review by New Zealand Health and Disability Ethics Committees (HDECs); therefore, formal ethics approval was not required.

## Consent

The patient and patient’s whanau (family) provided consent to be the subject of this case report and understood that she/her whanau could withdraw their consent at any time before presentation and/or publication of this report.

## Conflicts of Interest

The authors declare no conflicts of interest.

## Data Availability

The data that support the findings of this study are available from the corresponding author upon reasonable request.
